# Lymphoplasmacytic Lymphoma Presenting as Severe Secondary Raynaud’s Phenomenon

**DOI:** 10.7759/cureus.74144

**Published:** 2024-11-21

**Authors:** Drayton J Rorah, Salah Daghlas, Mashood Badshah, Paul Schmidt, Mehrdad Maz

**Affiliations:** 1 Internal Medicine, University of Kansas Medical Center, Kansas City, USA; 2 Division of Allergy, Clinical Immunology, & Rheumatology, University of Kansas Medical Center, Kansas City, USA

**Keywords:** clinical rheumatology, cryoglobulins, delayed diagnosis, diagnosis, digital gangrene, lymphoplasmacytic lymphoma, raynaud’s phenomenon, secondary raynaud’s phenomenon

## Abstract

Primary Raynaud’s phenomenon (RP) is a common and self-limiting condition, which is not secondary to any other disease process. In contrast, secondary RP has an underlying etiology. Several conditions can lead to secondary RP, which creates a challenging landscape for clinicians. Differentiation between primary and secondary RP is vital as failure to do so can lead to delays in treatment and poor patient outcomes. We present a case of a 77-year-old male who experienced digit discoloration when exposed to cold temperatures. He had been initially diagnosed with primary RP, but his symptoms had increased in frequency and progressed to digit necrosis requiring amputation. He was admitted to our tertiary care center for further workup. Subsequently, a diagnosis of type I cryoglobulinemia secondary to lymphoplasmacytic lymphoma (LPL) was made instead of the initial diagnosis of primary RP as the cause of his digit necrosis. This report emphasizes the importance of differentiating between primary and secondary RP and highlights the need for comprehensive workup in patients with RP, especially those presenting with atypical features.

## Introduction

Raynaud's phenomenon (RP) is a vasculopathy that manifests as episodic digital vasospasm, leading to pallor discoloration when exposed to stress and/or cold temperatures. Primary RP is a common and self-limiting condition, which is not secondary to another disease process [1]. In contrast, secondary RP develops due to an underlying etiology. Numerous conditions can lead to secondary RP, including hematologic, autoimmune, metabolic, and mechanical etiologies. We present a case of cryoglobulin-induced digital ischemia due to lymphoplasmacytic lymphoma (LPL), also known as Waldenström macroglobulinemia, initially thought to be primary RP.

LPL is a rare lymphoproliferative disorder associated with monoclonal production of IgM antibodies. It can be associated with type I cryoglobulinemia, which is characterized by excess production of monoclonal IgM antibodies. About 5.5% of symptomatic patients have cryoglobulinemia, and in 1.3% of patients, this may be the presenting feature, as was the case with our patient [2]. It is associated with acrocyanosis and RP, which may lead to gangrene if left untreated. This report highlights the clinical challenges faced when evaluating RP, especially in older males, as it is frequently associated with an underlying condition [3]. It is crucial to correctly identify whether RP is primary or secondary, as failure to determine the underlying etiology can lead to delayed diagnosis and treatment and, consequently, poor patient outcomes [4].

## Case presentation

The patient was a 77-year-old male who had begun experiencing digital discoloration and mild discomfort upon cold exposure, starting three years ago. He had a past medical history notable for coronary artery disease with ischemic cardiomyopathy, femoral artery stenosis with iliofemoral endarterectomy, and dyslipidemia. He had noticed purple discoloration of his upper extremity digits associated with tingling when working outside in the cold or handling cold objects. He had been evaluated by his primary care physician at a community health system and diagnosed with primary RP. 

His symptoms had remained persistent over the next two years, prompting a referral to a community rheumatologist and an autoimmune workup, which had been unrevealing. He had also been seen by a hematologist since 2020 for monoclonal IgM gammopathy. Three months before the current presentation, his symptoms had rapidly progressed with increased and persistent cyanosis of his digits. He had been evaluated by a vascular surgeon, with non-invasive vascular studies revealing mild non-obstructive atherosclerotic disease of the upper and lower extremities. The patient's digital purplish discoloration had progressed to digit necrosis requiring partial amputation of the left fourth and fifth digits. In September 2023, he had been noted to have splenomegaly, prompting a bone marrow biopsy. The biopsy had revealed a B-cell lymphoma with a MYD 88 mutation and a diagnosis of LPL. While in recovery from his recent partial amputations, he experienced a recurrence of digit gangrene, prompting referral to our tertiary care center. 

**Figure 1:**
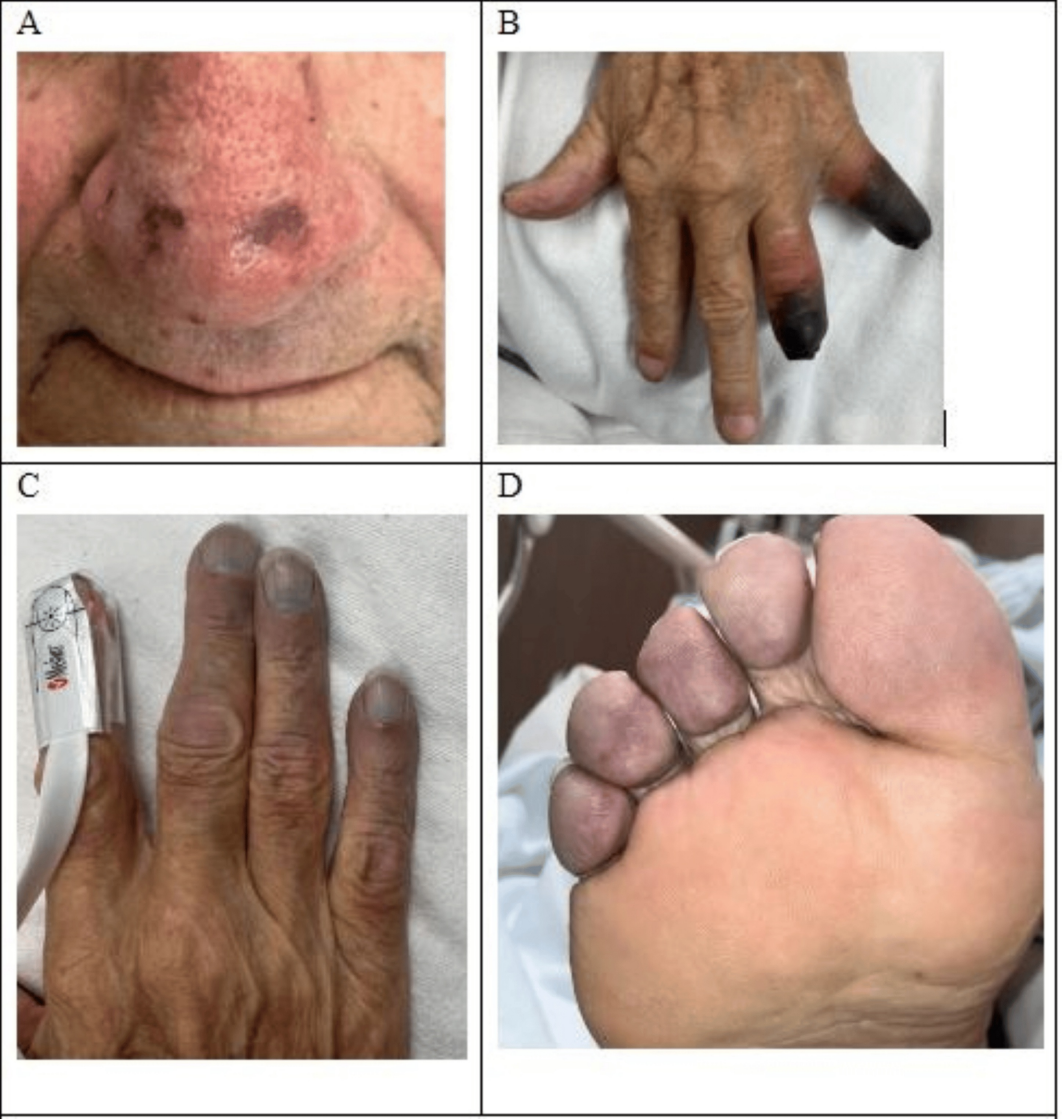
Exam findings of cyanosis and necrosis A: nasal tip cyanosis; B: dorsal left hand digit necrosis; C: dorsal right hand digit cyanosis; D: plantar right foot digit cyanosis

The patient presented to our academic hospital with multi-digit gangrene involving his left fourth and fifth fingers with additional acrocyanosis of his right fingers, right toes, and nasal tip (Figure 1). Rheumatology was consulted and serologic testing was performed (Table 1), which was notable for an elevated IgM and negative cold agglutin panel; however, the cryoglobulin panel revealed elevated cryoglobulin at 4% part per trillion (ref. negative) with positive monoclonal IgM kappa on immunofixation. This finding was consistent with type I cryoglobulinemia secondary to LPL rather than the initial diagnosis of primary RP as the cause of the digit necrosis.

**Table 1:** Serologic workup of the patient ANA: antinuclear antibody; CRP: C-reactive protein; ESR: erythrocyte sedimentation rate; RNP: ribonucleoprotein

Test	Result	Reference range
ANA screen	<80	<80 titer
Anti-Smith antibody	<0.2	<1 AI
Anti-RNP antibody	<0.4	<1 AI
Anti-SSA	<0.2	<1 AI
Anti-SSB	<0.2	<1 AI
Rheumatoid factor screen	13	<25 IU/mL
Cold agglutinins	<1:32	<1:32
Centromere antibody	<0.2	<1 AI
Myeloperoxidase antibody	<0.2	<1 AI
Serine protease 3 antibody	<0.2	<1 AI
Complement C3	101	88-200 mg/dL
Complement C4	<8.0	10-49 mg/dL
Jo 1 antibody	<0.2	<1 AI
SCL70 antibody	<0.2	<1 AI
Beta-2 glycoprotein 1 AB IgG	<1.4	<20 U/mL
Beta-2 glycoprotein 1 AB IgM	0.2	<20 U/mL
Anti-CCP IgG	<0.5	<3 U/mL
Cardiolipin IgG	<1.6	<20 GPL/mL
Cardiolipin IgM	<0.2	<20 MPL/mL
Dilute Russell's viper venom	1.1	<1.3 ratio
Hexagonal lupus anticoagulant	3s	Negative
IgG	536	762-1488 mg/dL
IgM	884	38-328 mg/dL
IgA	45	70-390 mg/dL
ESR	79	0-22 mm/hr
CRP	2.8	<1.0 mg/dL
Serum viscosity	1.2	≤1.5 cPoise
Cryoglobulins	4%	Negative
Immunofixation cryoglobulin	Type I (monoclonal IgM kappa)	Negative

Meanwhile, further progression of gangrene necessitated additional digit amputation of the left upper extremity along with sympathectomy by plastic hand surgery. The hematology team, in consultation with the rheumatology service, initiated LPL therapy with plasma exchange and high-dose steroids followed by zanubrutinib and rituximab. The patient was discharged home to continue outpatient chemotherapy. He subsequently required partial amputation of the third through 5th digits of his left upper extremity and the second through fourth digits of the right lower extremity. He continued to follow up with hematology and plastic surgery.

## Discussion

Primary RP is considered a benign vasospastic process, which often occurs following exposures to cold temperatures or emotional stress. Typical clinical symptoms include triphasic digital discoloration, which can be present in single or multiple digits. Patients often experience pale digits from decreased blood flow, followed by cyanosis from decreased blood flow to the tissue, which can be accompanied by numbness and tingling. This is followed by hyperemia due to vascular reperfusion of the tissue. RP symptoms typically resolve after a few minutes but can last a few hours in some cases. This exaggerated vasoconstriction is most frequently seen in young females between the second and fourth decades of life [5-7].

While primary RP is not caused by an underlying condition, certain comorbidities, such as diabetes mellitus and cardiovascular disease, may provoke attacks [7]. Although it is more prevalent in young females, primary RP can present in patients over 60 years of age [3]. In comparison, secondary RP refers to the presence of a vasospastic process in which an underlying disease causes vasoconstrictive episodes. Several conditions and diseases can alter the physiologic regulation of blood flow, resulting in RP. Autoimmune diseases such as systemic lupus erythematosus, Sjogren’s syndrome, and systemic sclerosis can cause secondary RP. Thromboangiitis obliterans (Buerger's disease) and certain hematologic conditions such as cryoglobulinemia, cold agglutinin disease, and paraproteinemia could also lead to this vasospastic process [8]. Exposure to medications such as beta-blockers, ergotamine, stimulants, or repetitive trauma of the palmar arteries as seen in construction workers can result in secondary RP [9,10]. Additionally, endocrinopathies such as hypopituitarism and hypothyroidism can lead to similar manifestations [11].

In contrast to secondary causes of RP, some conditions mimic RP, including peripheral neuropathy, acrocyanosis, paraneoplastic acral vascular syndrome, occlusive vascular disease, or excessive cold sensitivity [12-16]. Generally, these conditions have partial or complete physical exam findings similar to Raynaud’s, which further complicates the diagnostic accuracy. Distinguishing between primary and secondary RP and mimicking conditions is vital to determine which patients need additional workup to identify the underlying condition. Failure to do so could lead to delayed diagnosis, progression of disease, and morbidity as demonstrated in this case report. 

Several factors should increase a clinician's suspicion related to secondary RP. These include male sex, age greater than 40 years, severe attacks with prolonged ischemia, lack of a precipitating event, digital ulcerations, asymmetric attacks, proximal extremity involvement, abnormal nail fold capillaroscopy, and abnormal autoimmune laboratory serologies. Workup should begin with a comprehensive autoimmune review of systems and physical exam [17]. Review of medications including beta-blockers and stimulants, recreational drugs, and smoking history is a crucial part of the history taking and assessment. Nail fold capillary microscopy can be a useful tool, often utilized by rheumatologists to distinguish primary from secondary RP.

The presence of capillary dropout or distorted/enlarged capillaries could suggest an underlying rheumatologic disease process, specifically a connective tissue disorder such as scleroderma. Patients with an increased suspicion of secondary RP should undergo additional serological testing. Clinical history and physical examination should drive a focused workup based on the individual’s risk factors. However, a general workup includes, but is not limited to, complete blood count with differential, comprehensive metabolic panel, urinalysis with sediments, antinuclear antibody (ANA), rheumatoid factor (RF), thyroid stimulating hormone, erythrocyte sedimentation rate (ESR), C-reactive protein (CRP), serum protein electrophoresis, complement levels, cryoglobulins, antiphospholipid antibodies, and viral hepatitis serologies, particularly hepatitis C.

Vascular imaging can be useful in patients who exhibit symptoms such as asymmetric blood pressure/pulses or single-digit involvement to evaluate for large or medium vessel disease. Doppler ultrasound can be a useful cost-effective tool to delineate vasculitis versus vasculopathy. Angiography is an alternative option but is often reserved to determine if surgical intervention would be beneficial for macrovascular disease. Magnetic resonance angiography (MRA) is another modality available to evaluate for higher-quality evaluation, although, to our knowledge, no large studies have evaluated the use of MRA compared to Doppler ultrasound or conventional angiography. Lastly, laser Doppler imaging and thermal imaging are emerging tools to assess microcirculatory flow. This modality can assist in differentiating different conditions such as scleroderma from RP. However, these methods are not widely available in the clinical setting [18]. 

The management of RP symptoms depends on the severity and underlying etiology. If they are mild and do not cause discomfort to the patient, non-pharmacological management may be pursued, which usually entails avoiding triggers, minimizing cold exposure to extremities, and adequate stress control. If the patient experiences significant discomfort or pain, pharmacological options may be offered, most commonly long-acting dihydropyridine calcium channel blockers. Other options may include topical nitrate, phosphodiesterase type 5 inhibitors, angiotensin II receptor blockers, or serotonin-reuptake inhibitors. More severe cases, more commonly seen in secondary RP, may be treated temporarily with anticoagulation and/or intravenous prostaglandins. Refractory cases can be managed with sympathectomies, with a preference for digital over spinal. In addition, treatment of the underlying etiology in patients with secondary RP may help manage the symptoms [19]. 

Our patient was initially diagnosed with primary RP; however, he presented with many features that would be more consistent with secondary RP. His age, prolonged ischemia, and digital necrosis suggested a more insidious process. The onset of his RP had preceded the diagnosis of LPL by two years, which, in retrospect, could have been a period of intervention to prevent digit loss. As our report demonstrates, cryoglobulinemia may present as RP and progress to severe digital ischemia and necrosis if untreated. Cryoglobulins contribute to immune complex deposition, leading to vasculitis and subsequent vascular compromise [20]. The overlap in clinical features between primary RP and secondary RP and cryoglobulin-induced ischemia requires careful consideration, as failure to distinguish these two conditions could lead to poor patient outcomes.

## Conclusions

This report highlights the importance of distinguishing between primary and secondary RP. Several factors, such as age, sex, and severity of presentation, may point towards a secondary etiology. Clinicians should maintain a comprehensive and broad differential when managing patients with RP, ensuring timely identification of secondary causes and intervention for underlying pathology to optimize patient outcomes.
